# Improved dung beetle optimized MPPT with novel FOTPIDn(1 + PD) control in grid connected PV for optimal power sharing

**DOI:** 10.1038/s41598-026-43417-8

**Published:** 2026-04-25

**Authors:** Rachna Dhir, Rachana Garg, Uma Nangia

**Affiliations:** Department of Electrical Engineering, Delhi Technological University, Delhi, India

**Keywords:** Photovoltaic, Maximum power point tracking, Improved dung beetle optimization algorithm, Fractional order tilt proportional integral derivative, Total harmonic reduction, Energy science and technology, Engineering

## Abstract

The increasing demand for clean and sustainable energy has led to the widespread adoption of photovoltaic (PV) systems. This method proposes a novel bio-inspired control methodology to increase the performance of grid-connected PV systems under different climatic conditions. The article introduces a maximum power point tracking (MPPT) technique based on an improved dung beetle optimization (IDBO) algorithm for efficient power extraction and inverter management. For the population diversity, the IDBO algorithm uses a multi-strategy mechanism, like Fuch chaotic mapping. The N-Filter, a one plus proportional derivative (1 + PD) controller, and a fractional order tilt proportional integral derivative (FOTPID) controller are designed to improve the inverter’s performance even more. The difficulties in adjusting the controller setting in a non-linear and erratic grid tied PV system are addressed by the suggested hybrid controller. The IDBO algorithm optimizes the inverter control parameters to minimize direct current (DC) link voltage error and total harmonic distortion (THD) in output voltage and current. The simulation outcomes exhibit that the proposed approach expressively improves the power quality and dynamic response under varying environmental conditions. During the irradiance variation, the system attains a steady state power output of 7723.613W, with a peak power of 7741.121 W at 0.397 s, a settling time of 0.049 s, and a minimal overshoot of 0.22%. Under the temperature variation, the steady state output reached 7007.978 W with a peak response time of 0.088 s, settling time of 0.216 s, and an overshoot of 8.316%. This work also provide the mean squared error (MSE) of 0.046, root mean squared error (RMSE) of 0.215 and mean absolute error (MAE) of 0.175. This result confirms the supremacy of the proposed IDBO algorithm optimized control strategy in maintaining system stability, reducing DC-link voltage error, minimizing THD, and improving the overall performance of the grid connected PV system.

## Introduction

The request for energy has increased over the years, driven by industrial growth, technological advancements, and population expansion^1^. Renewable energy resources (RER) are a necessary alternative to traditional energy sources that have satisfied these demands in the

past, such as coal and oil, due to their limited availability and negative effects on the environment^2^. High irradiance, broad availability, and cheap operating costs have propelled solar PV schemes to the forefront of this category^3,4^. Air temperature fluctuations and uneven irradiance are two external conditions that limit the power output of PV systems and, consequently, their efficiency^5^. A DC-DC converter is used to increase the DC output power of solar photovoltaics, which is inadequate for some uses. The DC-DC converter’s primary function is to track the MPP as it relates to the I-V curve^6^. Regardless of variations in temperature and irradiance, the operating system may use MPPT techniques to increase the solar system’s efficiency at MPP on the PV curve^7^. To address the difficulties of pursuing the global maximum power point (GMPP) while reducing inefficiencies due to local maxima, advanced MPPT solutions have been suggested. Inefficient energy harvesting is a common consequence of using old-school MPPT methods like Incremental Conductance and Perturb and Observe (P&O)^8,9^.

An inverter’s typical function is to change the voltage of an electrical circuit from DC to alternating current (AC). In order to generate electricity from renewable sources, power converters are required, and the whole system of power converters needs to be reliable and efficient^10^. An inverter is a common interface for solar systems^11^. To ensure compliance with grid standards and system frequency, an inverter’s primary job is to control the voltage and current flow of power from renewable sources to the grid^12^. Grid connection requirements are the primary determinants of the current–voltage phase angle^13^. The grid-side controller consists of an exterior voltage loop and an inner current loop. Because of its quick dynamics, the inner loop is in charge of grid synchronization^14^. Controlling reactive power, protecting the system, and enhancing power quality are the goals of the inner loop. This inner loop alone produces the reference signal for pulse width modulation (PWM). In order to send the most extracted power to the grid, the external voltage loop, which is fed forward, aids in maintaining a steady DC link voltage (Vdc)^15^. The power fluctuations may be seen as a disruption that affects power quality and DC-link voltage control. Typically, the DC-link voltage is adjusted using a feedback controller to maintain unity power factor operation, even when faced with model uncertainty or external disturbances^16,17^.

Several approaches have been proposed for grid-connected power converters with the goals of achieving global stability and good transient performance. The writers also provide a variety of solutions for attainment the most energy out of PV panels. When it came to extracting the most energy possible from MPPT-based PV systems, the author in^18^ turned to salp-swarm optimization. Optimization of the PV system by the Emperor Penguin under varying irradiation conditions is discussed in^19^. The author^20^ utilized the horse herd optimization for extracting the maximum power based on different weather conditions. Although numerous control and optimization techniques have been developed for grid-connected PV systems. Conventional MPPT methods struggle to track the global maximum power point under partial shading and dynamic environmental conditions. Even advanced metaheuristic algorithms face difficulty while improving tracking capability, such as premature convergence, local optima stagnation, parameter sensitivity and inconsistent performance under dynamic operating scenarios.

Many existing studies focus either on enhancing MPPT performance or improving inverter control independently, lacking a coordinated framework. Consequently, this study offered a new method to reduce harmonic distortions and oscillations, which improves performance in different environmental conditions and provides a solution for the renewable energy industry. The objectives of the proposed system are given below.

- To develop and implement a novel bio-inspired control strategy for the grid-connected PV system to augment MPPT performance and expand inverter control under varying conditions.
- To achieve improved performance by using an IDBO algorithm with fuch chaotic mapping for the population diversity and the hybrid controller with the filter.
- To minimize the DC-link voltage error and THD for robust performance during the rapid variations in the irradiance and temperature.

The organization of this paper is described as follows: Section "Related works" describes the various related works based on MPPT. The suggested scheme is comprehensive in Section "Proposed methodology", which covers the modeling of the PV system, battery, IDBO algorithm based MPPT control, and FOTPIDn(1 + PD) controller. The simulation outcomes are described in Section "Modeling of photovoltaic panel". Finally, a conclusion with future scope is delivered in Section "Modeling of battery".

## Related works

Some of the existing works related to renewable sources integrated grid connected systems with different control strategies are discussed in this section. By utilizing a fuzzy self-tuning controller (FST) to update the proportional integral derivative (PID) controller, Ibrahim et al.^21^ introduced a new approach to adaptive MPPT for grid-connected photovoltaic systems, which improves upon the traditional incremental conductance (INC). By linking a grid-connected inverter to the DC output of the PV, the INC-FST allows for dynamic adjustment of the boost converter signal. To calculate the effect of a voltage variation, the controller senses small changes in the PV array’s voltage and current using INC algorithm. Outputs from the controller are the gains, whereas inputs to fuzzy self-tuning are the error and the change of error. This method obtained a high efficiency of 99.80% under partial shading conditions. In order to achieve MPPT in a PV system that is connected to the grid, EL-Banna et al.^22^ presented a golf optimization Approach (GOA). In order to find the best inverter features for the voltage and current regulators, the same method was also used. A method for lowering the output voltage and current’s DC voltage error and total harmonic distortion is the focus of this effort. The outcomes show that the suggested technique can improve the tracking system and the utility grid’s power quality under a variety of partial shade effects and temperature changes. The suggested method attains a 99.8% tracking efficiency.

Mazumdar et al.^23^ suggested a grey wolf optimized (GWO) fractional order proportional integral derivative (FOPID) controller for grid connected system. The controller was intended to draw as much energy as possible from the solar source in order to optimize electricity. The suggested FOPID controller has the adaptive nature of a PID controller and optimizes its gain parameter, taking into account elements on both the generator and grid sides.

The suggested research optimized the FOPID controller with grey wolf optimization (GWO) to maximize power, control the DC link’s voltage and current, and follow the model’s quadrature axis. Huang et al.^24^ introduced a fractional controller for three-phase photovoltaic inverters based on the enhanced Oustaloup algorithm for fractional modelling, and control in the grid-connected solar inverter system. The simulated annealing process was used in the numerical optimization method for parameter tuning of the voltage outer loop fractional PIλ controller, with integral time absolute error (ITAE) serving as the optimization performance index. The findings showed that a fractional controller for a three-phase PV inverter system based on an extended Oustaloup algorithm may reduce harmonic pollution, improve dynamic and static characteristics, and significantly improve grid-connected power quality.

Altawil et al.^25^ successfully controlled the active and reactive power of solar inverters using fractional order proportional-integral (FO-PI) controllers and a salp swarm optimization method (SSA). These controllers can control the active and reactive power of solar inverters by tapping into their reactive power absorption and production capabilities. This could fix problems with overvoltage and undervoltage as well as keep the voltage at the point of common coupling (PCC). For grid-connected PV systems with an integrated multi-level inverter, Boucheriette et al.^26^ suggested using an FOPI controller to boost efficiency. Two control loops based on FOPI manage the direct current (Id) and quadratic current (Iq) of the multilayer inverter, while a third loop controls the intermediate circuit voltage (Vdc). Improving the dynamic behavior of grid- connected PV systems is the main goal of this work. In this study, the FOPI Controller’s gain parameters are adjusted by means of the GWO technique. Table 1 represents the comparative analysis of existing works in terms of methods, controllers utilized, advantages, and disadvantages.

**Table 1:** Comparative analysis of existing works.

Author	Algorithm	Controller	Advantages	Disadvantages
Ibrahim et al.^21^	INC-FST	PID with Fuzzy Self- Tuning	Achieves excellent MPP tracking performance, especially under partial shading conditions	Requires careful design and tuning of the fuzzy logic system, which can be time-consuming
EL-Banna et al.^22^	GOA	PI	Effectively minimizes DC voltage error and reduces harmonic distortion	Potential for slower convergence compared to other methods
Mazumdar et al.^23^	GWO	FOPID	Optimized for generator and grid conditions	Less accurate
Huang et al.^24^	Simulated Annealing	Fractional PIλ	Reduced harmonics, improved dynamic/static characteristics	Complexity of fractional order control implementation
Altawil et al.^25^	SSA	FOPI	Reduced harmonic distortions	Complexity of FOPI controller implementation and tuning
Boucheriette et al.^26^	GWO	FOPI	Improved system stability by regulating reactive power flow and mitigating voltage fluctuations	High computational overhead

Although significant advancements have been made in grid-connected PV systems through intelligent optimization algorithms and fractional-order controllers, several research gaps still remain. Existing works such as INC-FST, GOA, GWO-FOPID, SSA-FOPI and fractional controllers primarily focus on improving MPPT efficiency. Here many metaheuristic based methods suffer from limitations such as slow convergence speed, local optima stagnation, high computational complexity and difficulty in parameter tuning dynamic environmental. Fractional-order controllers, although effective in harmonic reduction and dynamic improvement, they also involve in complex implementation and increased design burden. Therefore there is a clear need for advanced optimization based hybrid control method is required.

## Proposed methodology

This research work proposes a novel improved dung beetle optimization based MPPT technique for grid tied PV systems that aims to effectively manage multiple power peaks and optimize overall power output. First, the PV system generates electricity, and then, with the aid of optimization-based MPPT, the maximum power is monitored. Then, an N-filter with a FOTPID (1 + PD) controller is recommended for optimal inverter tuning. Because PV power delivered by grid-connected systems is non-linear and subject to climatic circumstances, it is challenging to choose the PID controller parameters for MPPT. The inverter’s voltage and current regulating mechanism’s optimal features are determined using the proposed optimization algorithm. A method for lowering the output voltage and current’s DC voltage error and total harmonic distortion is being evaluated. Consequently, the FOTPIDn(1 + PD) controller’s parameters are fine-tuned using an upgraded dung beetle optimization technique. It is well known that the normal DBO is not very accurate and often gets stuck in local optima. IDBO algorithm is suggested to deal with these problems. To begin, the dung beetle population is seeded with more individuals and more variety using Fuch chaotic mapping. This improved algorithm, which finds the best mix between exploring and exploiting, makes sure that the search for optimal solutions works well. By using the IDBO algorithm to find the best settings for the inverter based on the grid’s power quality, the total harmonic distortion of the grid stays within the acceptable range.

**Fig. 1:**
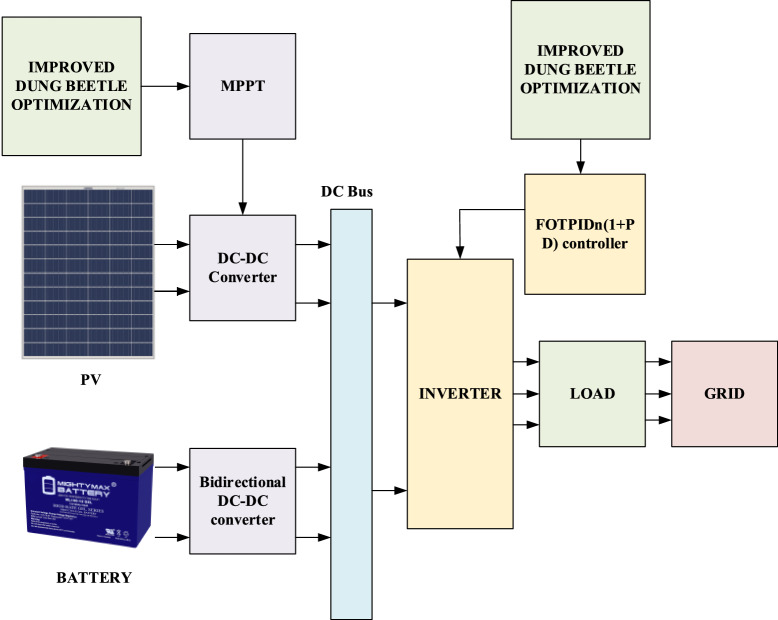
Block diagram for the proposed method.

**Fig. 2:**
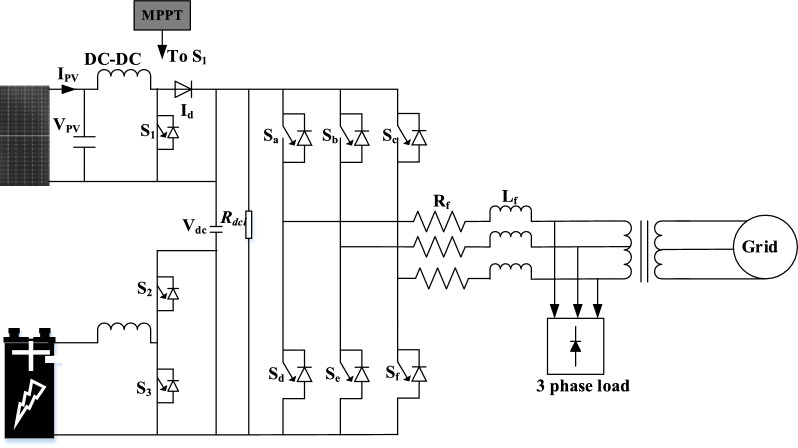
Equivalent circuit for the proposed methodology.

The system’s total power quality improves when the inverter’s settings are optimized. Here, Fig. 1 represents the block diagram, and Fig. 2 illustrates the circuit diagram for the suggested approach.

Here the Fig. 2 represent the grid connected PV for power generation with the DC-DC converter, battery storage and a three-phase inverter. The PV array gives DC power and MPPT controller with boost converter is used to extract the maximum power point. The boost converter therefore stabilizes and increases the PV $V_{PV}$ voltage to maintain a stable DC voltage. The battery storage is incorporated through a bidirectional switching device to support the power balance.

The DC link $V_{dc}$ capacitor minimizes the voltage ripple and provide stable input to the three phase VSI with 6 switches from $\left( {S_{a} - S_{f} } \right)$. This is used to convert the DC power to the AC for the suitable grid connection. The $R_{f} L_{f}$ filter is obtained at the output of the inverter side to reduce the harmonic distortion. Moreover, this also increases the power quality before supplying to the 3 phase load and the grid.

## Modeling of photovoltaic panel

**Fig. 3:**
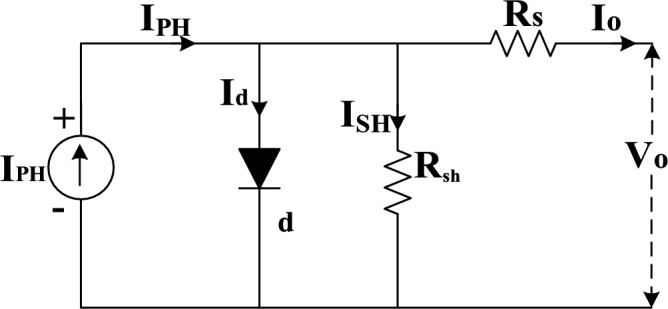
PV circuit module.

A semiconductor-based energy conversion device, a PV panel, directly changes sunlight into electricity through the PV effect^27,28^. An analogous current is generated by a photocurrent source, and the non-linear behavior of the PN junction of the solar cell is described by the diode. In a cell, the shunt resistance shows how current leaks out of the cell. In contrast, the series resistance takes into consideration the voltage drop caused by the material’s and connections’ internal resistance^29^. This design strikes a perfect compromise between computational simplicity and accuracy, making it excellent for regulating and analyzing the performance of both independent and grid-connected PV systems. Figure 3 represents the PV circuit module.

As shown in Fig. 3, the PV cell’s behavior can be replicated by connecting a current source in series with a diode, a shunt resistance, and a series resistance; these components stand in for the cell’s intrinsic features and power losses, respectively. The disparity between the photo-generated 

current I_PH_ and the one stated by Kirchhoff’s current law (KCL) and the sum of the diode current.

I0 , shunt current, and voltage drop across the series resistance Rs equals the total curren I produced by the PV cell . When the KCL is applied at the circuit’s output node, the PV current source is represented by Eqs. (1) and (2), which give the subsequent condition for the PV cell output current.

1 $$I_{PH} = I_{d} + I_{SH} + I$$

2 $$I = I_{PH} - I_{SH} - I_{d}$$

3 $$I = I_{PH} - I_{0} \left[ {\exp \left( {\frac{{V + IR_{s} }}{{nV_{t} }}} \right) - 1} \right] - \left[ {\frac{{V + IR_{s} }}{{R_{sh} }}} \right]$$

Here, Eq. (3) represented the current value for the PV system. In the PV cell module, here.

I0 stands for the diode reverse saturation current, *n* characterize the ideality factor, and *V* for

the system voltage output. *R*_*sh*_ represent the shunt resistance, R_s_ represent the series resistance, I depicts the output current, I_PH_ depicts the PV current, *I*_*d*_ represent the diode current and *I*_*SH*_ represent the shunt current. After the solar panel generates the electricity, an efficient system is needed to harvest and control that power. This is accomplished by raising the voltage level from the panel to the intended output using the boost converter.

## Modeling of battery

An integral part of a photovoltaic system, batteries store the electrical energy generated by the solar panels for usage at a later time^30^. The Tremblay technique faithfully captures dynamic charging processes that are commonly used to describe battery behavior in the PV system. These batteries can store energy during times of ample generation and use it when solar output is low or demand is high. They operate effectively within certain voltage and current limits to ensure reliable performance and system stability^31^. In a standalone hybrid system, the charge and discharge mechanism particularly guarantees the stability and reliability of the total PV scheme.

4 $$V_{0} = B_{bat} - R_{{\mathrm{int}}} I$$

5 $$B_{bat}^{char} = B_{0} - K\frac{B}{{I_{T} - 0.1E}}I^{ \otimes } - K\frac{B}{{E - I_{T} }}I_{T} + Ae^{{( - BI_{t} )}}$$

6 $$B_{bat}^{disch} = B_{o} - K\frac{B}{{B - I_{I,T} }}(I_{T} + I^{ \otimes } ) + Ae^{{( - BI_{t} )}}$$

7 $$SOC(\% ) = SOC(\% ) - 100\left( {\frac{{\int {idt} }}{B}} \right)$$

The battery charging and discharging are represented in Eqs. (4) to (7) $B_{bat}^{char}$ and $B_{bat}^{disch}$ in their capacitance is denoted as $B$, the filtered current is characterized as $I^{ \otimes }$, the polarization constant is depicted as $K$ , and the exponential voltage in the system is represented as $e^{{( - BI_{t} )}}$. The output voltage is denoted $V_{0}$, the battery current by $I_{T}$ , and the actual battery charge by $\int {idt}$. $A$ represents the exponential zone amplitude and SOC represent the state of charge of the battery. In contrast to the traditional method, the bidirectional converter allows electricity to travel both from the PV panel to the battery for charging and from the battery to the load grid during discharge ^33^.

## DC-DC converter

Installing a DC-DC step up converter between the PV panel and the load allows for the effective extraction of the maximum power from the PV panel.

To optimize the impedance between the panel and the load, the PV system needs this converter so it can operate close to its MPPT ^32^. In order to meet the greater voltage requirements of the storage system, the boost converter raises the input voltage of the PV panels.

8 $$V_{o} = V_{i} \frac{1}{1 - d}$$

9 $$I_{o} = I_{i} (1 - d)$$

here *Vo* and *Io* represent the boost converter output for both the voltage and current, *Vi* and *I*_*i*_*.*

represent as the input for both the voltage and current, and *d* represented as the boost converter duty cycle.

## Proposed MPPT techniques

MPPT is an important method for photovoltaic systems that ensures the panels operate at peak efficiency. In response to dynamic environmental conditions, such as temperature and solar radiation, this continuously adjusts the system’s operating point to optimize power output. The maximum power is provided, but the variance remains throughout the day, due to the solar panel’s non-linear voltage-current (V-I) characteristics. Without the MPPT, the PV system may run at suboptimal locations, which could result in significant power losses ^34^. The MPPT-based algorithm dynamically adapts the PV panel’s operating voltage and current in order to continually locate and operate at the MPP. In order to efficiently track and extract the extreme power from the PV system under a change of climatic conditions, the IDBO algorithm is used in this work to fine- tune the MPPT controller. In the IDBO algorithm, a fuch chaotic mapping is used to improve the dung beetle optimization so that this can give their maximum efficiency. Here, the fitness function for the MPPT is given in Eq. (10),

10 $$P_{PP} = V_{VV} \times I_{II}$$

In the MPPT section, the output power of the PV system $P_{PP}$, $V_{VV}$ characterize the output voltage of the PV and $I_{II}$ epitomize the output current of the PV system. This encourages quick settling and lowers long-term error by penalizing errors over time and giving greater weight to those that remain longer.

## Proposed multi stage FOTPIDn(1 + PD) controller

The FOTPIDn(1 + PD) controller is a strong and flexible solution for the non-linear, time varying system, such as those in power electronics, by combining the advantages of the FOTPID controller with a high order structure and a proportional derivative (PD) improvement ^35^ ^36^. By permitting the use of non-integer fractional order in the integration and differentiation, this hybrid controller design improves the fine tuning capabilities for a variety of system responses and provides more control dynamics flexibility. The N-filter of the FOTPID modules helps reduce noise sensitivity and overshoot, while the additional (1 + PD) structure improves transient performance, especially at high speed response settings.

The (1 + PD) element unity gain terms ensure the removal of steady state errors, which are typically an issue for PD controllers, particularly in low frequency working environments. Furthermore, the PD element’s predictive nature makes it easier to take early corrective action that guarantees that the voltage regulation and the current regulation happen faster. Additionally, the unity term promotes enhanced resilience to the parametric uncertainty and load disruptions. Here ITAE is used in the time domain performance index that are used to solve and optimize control systems. This penalizes the error that persists longer in time more heavily than the transient one, which gives a fast and stable region. Here, the ITAE equation for the controller circuit is given in Eq. (11),

11 $$ITAE = \int_{0}^{\tau } {t\left| {e(t)} \right|}$$

12 $$MSE = \frac{1}{N}\sum\limits_{z = 1}^{N} {\left( {Y_{z} - \hat{Y}_{z} } \right)}^{2}$$

13 $$RMSE = \sqrt {\frac{1}{N}\sum\limits_{z = 1}^{N} {\left( {Y_{z} - \hat{Y}_{z} } \right)}^{2} }$$

14 $$MAE = \frac{1}{N}\sum\limits_{z = 1}^{N} {\left| {Y_{z} - \hat{Y}_{z} } \right|}$$

Here *t* is the time, *e*(*t*) represents the error amongst the reference and actual output, and $\tau$

represents the total simulation time. Here in Eq. (12–14) $Y_{z}$ represent the actual value, $\hat{Y}_{z}$ represent the predicted value and $N$ represents the total number of observations.

The FOTPIDn(1 + PD) controller performs better in the DC-DC converter because it provides excellent voltage regulation, lower THD, and boosts the scheme efficiency. They are appropriate for the grid connected renewable energy scheme that has the capacity to react dynamically to the changing demand circumstances while preserving steady functioning. When combining with a clever optimization method such as the IDBO procedure that effectively adjusts the fractional strictures and gain settings for the peak performance, the controllers are extremely beneficial.

15 $$A_{FOTPIDn} (s) = \frac{Y(s)}{{E(s)}} = K_{P1} + K_{T} .s^{{\frac{ - 1}{n}}} + \frac{{K_{I} }}{{s^{\lambda } }} + K_{D1} .\frac{1}{{s^{\mu } }}.Ns$$

16 $$A_{(1 + PD)} (s) = \frac{Y(s)}{{E(s)}} = 1 + K_{P2} + K_{D2} .s$$

Here, Eqs. (15) and (16) represent the controller equations for the FOTPIDn and the (1 + PD) controller.

**Fig. 4:**
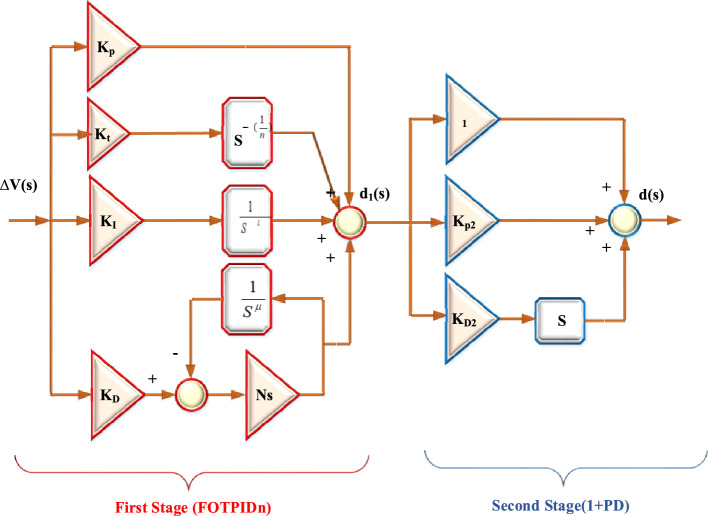
Controller block for the proposed method.

$K_{P1}$ and $K_{P2}$ characterize the proportional part for the two controllers, $K_{T} .s^{{\frac{ - 1}{n}}}$ represent the tilt parameter for the given device, $\frac{{K_{I} }}{{s^{\lambda } }}$ represent the integration part for the parameter, $Ns$ represent the filter parameter, $K_{D1} .\frac{1}{{s^{\mu } }}$ and $K_{D2} .s$ represent the derivative part for the two controller circuits. Equation (14) represents the total controller of the FOTPID(1 + PD) controller. Figure 4 represents the controller block for the suggested method.

17 $$A_{FOTPIDn(1 + PD)} (s) = \frac{Y(s)}{{E(s)}} = K_{P1} + K_{T} .s^{{\frac{ - 1}{n}}} + \frac{{K_{I} }}{{s^{\lambda } }} + K_{D1} \frac{1}{{s^{\mu } }}.Ns + 1 + K_{P2} + K_{D2} .s$$

The IDBO procedure was created and used to get around the complicated process of adjusting several non-linear and fractional order parameters. While employing Fuch’s chaotic mapping for the chaotic initialization, the IDBO algorithm improves on the conventional dung beetle optimization. These guarantee a more complete investigation of the solution space that prevents premature convergence and greatly boosts

population variety. Here $K_{P1}$, $K_{T} \cdot s^{{\frac{ - 1}{n}}}$ $\frac{{K_{1} }}{{s^{\lambda } }}$ $K_{D1} \frac{1}{{s^{\mu } }}$, $K_{P2}$, $K_{D2} \cdot s$* K*_*D*2_.*s* are used as the parameter for the optimization to tune the controller.

Using the hybrid nature of the IDB algorithm, the controller’s tilt, filter, integral, proportional, and derivative parameters are adaptively modified to lower the objective function, including THD, overshoot, and steady state error. In order to expand the proposed controller for both transient and steady state performance under various environmental and load conditions, the algorithm dynamically balances the exploration and exploitation phases.

## Improved dung beetle optimization algorithm

**Fig. 5:**
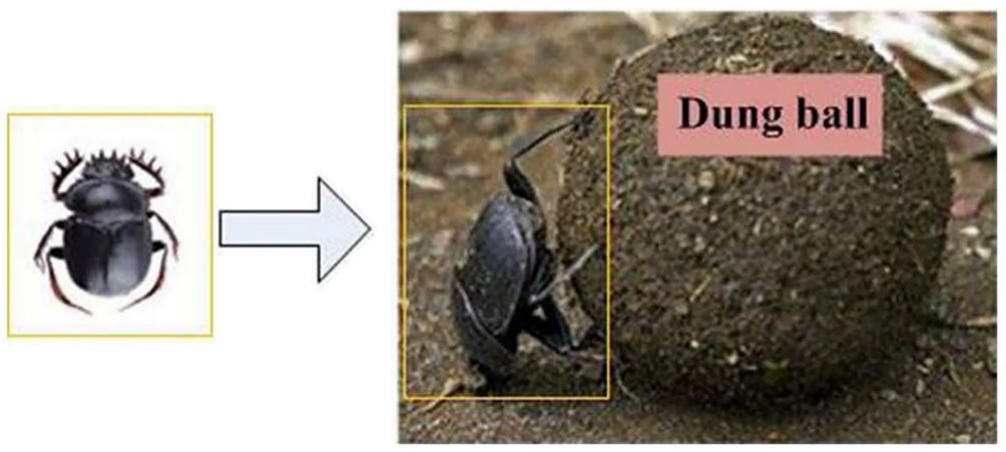
Dung beetle rolling dung ball.

The dung beetle optimization method (DBO), which was encouraged by an optimization technique from 2022, was introduced by Jianka Xue and Bo Shen. The clever and adaptable actions of the dung beetle that have been seen in the wild, which include ball-rolling, dancing for orientation, foraging, thieving, and reproducing, serve as the foundational module for this algorithm. In both the exploration and exploitation stages, the optimization uses this behavior to convert into a mathematical operation in the direct search mechanism ^37^. Each of the four beetle species that the DBO algorithm separates the population into is represented as a distinct survival strategy that improves the total functionality of the program. Figure 5 represents the dung beetle rolling a dung ball.

## Rolling dung beetle

These beetles mimic the behavior of the worldwide search. This step guarantees that the search agent investigated a variety of areas of the solution space by simulating how dung beetles roll dung balls in a conventional line and adjust their trajectory response to astronomical signals in Eq. (18).

18 $$\left\{ \begin{gathered} A_{i} (L + 1) = A_{i} (L) + \alpha \times k \times A_{i} (L + 1) + b \times \Delta A \hfill \\ \,\,\,\,\,\,\,\,\,\,\,\,\,\Delta A = \left| {A_{i} (L) - l^{\omega } } \right| \hfill \\ \end{gathered} \right.$$

here *L* and *Ai* (*L*) depicts the present amount iteration, the location of the *i*^*th*^ dung beetle at the *L*

repetition, and the global worst location correspondingly.l^w^ represents the global worst location

and Δ*A* represent to simulate of the change in the global worst position.

##  Dancing dung beetle

This tendency is employed in DBO to improve directional reevaluation of the existing solution and keep the procedure from being trapped in the local optima. The dancing dung beetle stage that

updates the position of the solution by investigating new directions based on the combination of randomization and directional memory is activated when the algorithm identifies stability in fitness across iterations in Eq. (19).

19 $$A_{i} (L + 1) = A_{i} (L) + \tan (\theta )\left| {A_{i} (L) - A_{i} (L - 1)} \right|$$

##  Breeding dung beetle

These beetles naturally excavate tunnels beneath dung heaps and lay their egg in the dung balls. The larvae are able to develop in these protected breeding chambers. Similar to concentrating on the favorable areas in an optimization landscape, this behavior indicates stable nurturing and resource rich settings in Eq. (20).

20 $$C_{i} (L + 1) = l^{ * } + b_{1} \times (C_{i} (L) - Tb^{ * } ) + (C_{i} (L) - ub^{ * } )$$

The bottom and upper limits of the spawning area in this case are shown by Tb and ub respectively. . On the other hand, C gives exact details about location of egg where the t iteration is done.

##  Little dung beetle foraging for food

These act as fresh sources of solutions to preserve population variety. The larvae in Eq. (21) are stimulated to develop into adult beetles by employing random modifications that help them escape local optima and increase resilience.

21 $$A_{i} (L + 1) = A_{i} (L) + D_{1} \times (A_{i} (L) - Tb^{b} ) + D_{2} (A_{i} (L) - ub^{b} )$$

here *Ai* (*L*) , *Tb*^*b*^ and *ub*^*b*^ denote the precise details of the placement of *i*^*th*^ dung ball. The lower and higher.

limits of the foraging region are where the little dung beetle is found during the *L* iteration.

##  Stealing a dung beetle

Through this adoption of positive attributes from others, this component represents a big leap in the search space. Similar to stealing a better key component from the population elite, these bugs figuratively steal promising dung balls in Eq. (22).

22 $$A(L + 1) = l^{b} + S^{\prime} \times g^{\prime} \times \left( {\left| {A\left( L \right) - l^{*} } \right| + \left| {A\left( L \right) - l^{b} } \right|} \right)$$

Here *Ai* (*L*) , *l*^*b*^ denotes the precise details on the place of the *i*^*th*^ thieving dung beetle during the

*L* repetition as well as the optimal location of the result on the ongoing optimization.

##  Improved dung beetle optimizer

The original DBO is a strong metaheuristic procedure that is renowned for its simplicity and quick convergence. This was encouraged by the dung beetle’s natural activities, which include ball rolling, dancing, and breeding. However, as iteration progresses, population variation tends to decrease, making it vulnerable to trapping in local optima

and premature convergence. In the complicated search space, this restriction frequently lowers the algorithm’s accuracy and efficacy. To address these issues, an IDBO method is suggested. By increasing population variety and exploration capabilities, the fundamental DBO algorithm’s performance is improved during the initialization phase by using the Fuch chaotic mapping. The chaotic maps provide beginning populations that are very diverse and evenly distributed, in contrast to random initialization. Early in the optimization process, fuch mapping, in particular, improves global search capabilities by introducing controlled randomness and complexity. The procedure’s capacity to travel a larger solution space and prevent premature convergence is enhanced by a chaotic start. As a result, the algorithm maintains a more diverse population throughout the iterations, which increases the global optimum accuracy and convergence speed. Retaining diversity and fine-tuning the population distribution make the original DBO algorithm more fit for tackling complex, non-linear optimization problems, and the suggested IDBO algorithm substantially increases its efficiency. The mathematical equation of the Fuchs chaotic mapping is given in Eq. (23),

23 $$x_{n + 1} = \sin (\pi xn ) + \mu xn (1 - x_{n} )$$

where Eq. (23) is substituted in Eq. (18)

24 $$A(L + 1) = l^{w}$$

Now,

25 $$\left\{ \begin{gathered} A_{i} (L + 1) = A_{i} (L) + \alpha \times k \times A_{i} (L + 1) + b \times \Delta A \hfill \\ \,\,\,\,\,\,\,\,\,\,\,\,\,\Delta A = \left| {A_{i} (L) - \sin (\pi x_{n} ) + \mu x_{n} (1 - x_{n} )} \right| \hfill \\ \end{gathered} \right.$$

**Fig. 6:**
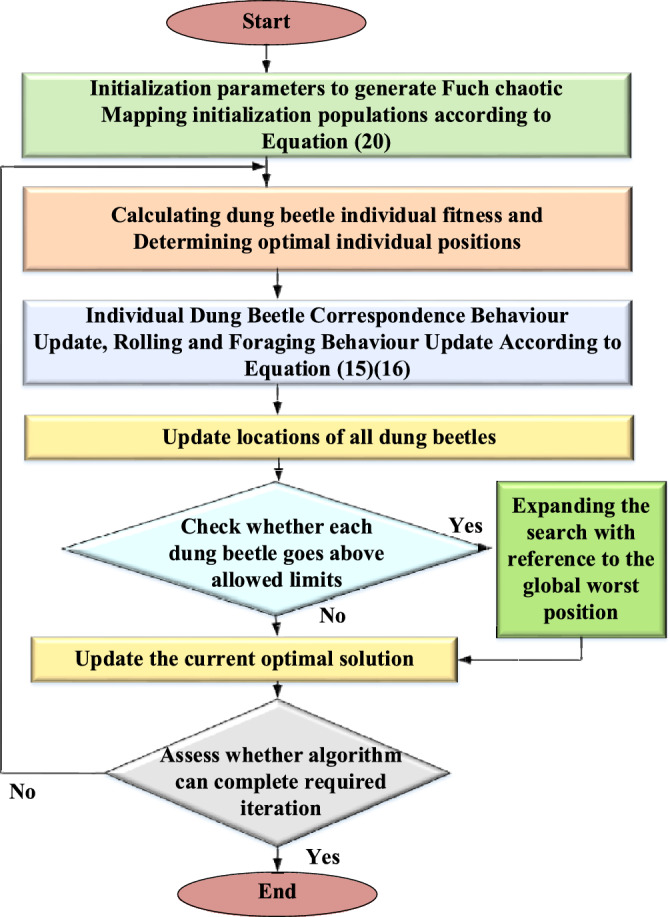
Flow chart of IDBO algorithm.

Equation (24–25) represents population variability in the IDBO algorithm, which enhances the global search capacity following the initialization of the population using the Fuch chaotic mapping. By strengthening the procedure, the capacity to break out of the local optimal and preventing premature convergence, this enhances quicker and more precise convergence towards the optimal solution. Figure 6 represents the flow chart for the IDBO algorithm.

## Result and discussion

**Fig. 7:**
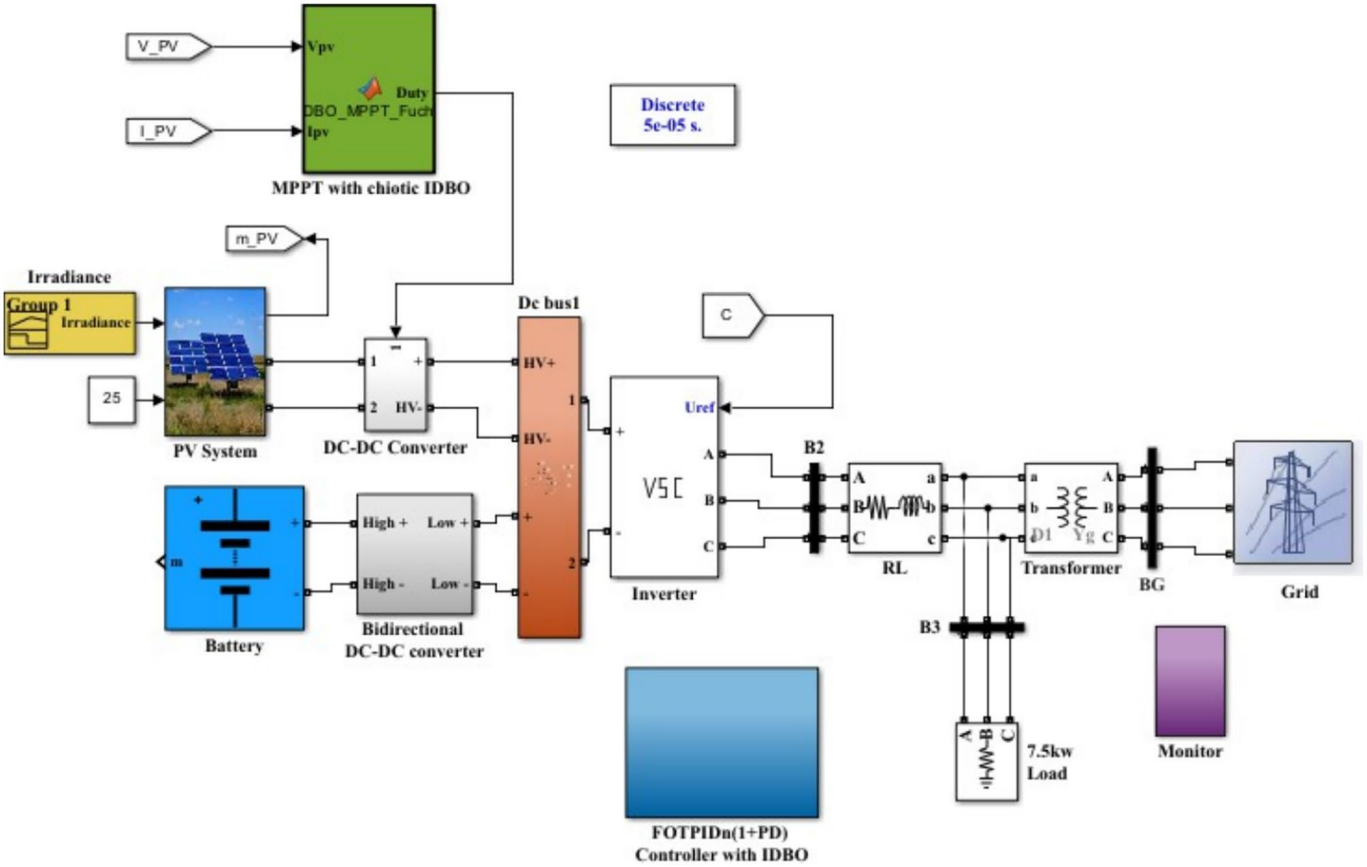
Simulink block diagram for the novel work.

The simulation result and performance assessment of the suggested MPPT controller based on IDBO algorithm and the integration of the FOTPIDn(1 + PD) controller is installed on the grid tied PV system are shown in this section. The whole module was developed and replicated using MATLAB/Simulink in order to assess the effectiveness of the suggested methodology. Figure 7 is the Simulink work for the novel operation for the proposed method. Table 2 represents the parameters for the different devices

**Table 2:** Parameters for different devices.

Parameter	Value
Photovoltaic	
Maximum power	7.8 kW
Cells per Modules	72
Open circuit voltage	110 V
Short circuit voltage	9A
Maximum power point voltage	102 V
Maximum power point current	8.5A
Coefficient of temperature-Voc	-0.36
Coefficient of temperature-Isc	0.04
Parallel string	3
Series connected module per string	3
Battery	
Nominal voltage	240 V
Rated Capacity	130.43Ah
Initial state of charge	50%
Battery response time	1 s
Converters	
FET resistance	1e-1
Inductance	250e-6
Internal diode inductance	0H
Internal diode voltage	0 V
Snubber resistance	10Ω
Snubber capacitance	15F
Load	
Phase to phase nominal voltage	300Vn
Active power	7.5 kW

**Fig. 8:**
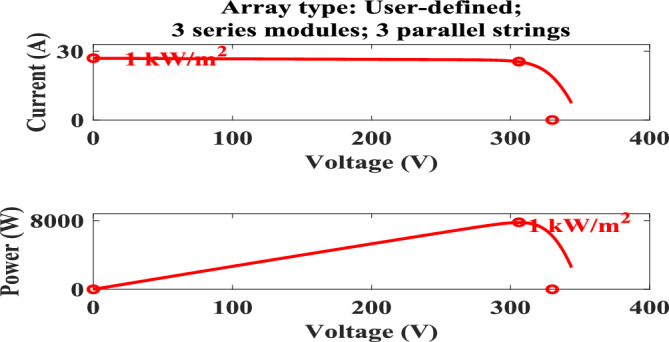
Analysis of constant stable condition for power and current.

**Case 1:** Constant Input Condition.

**Fig. 9:**
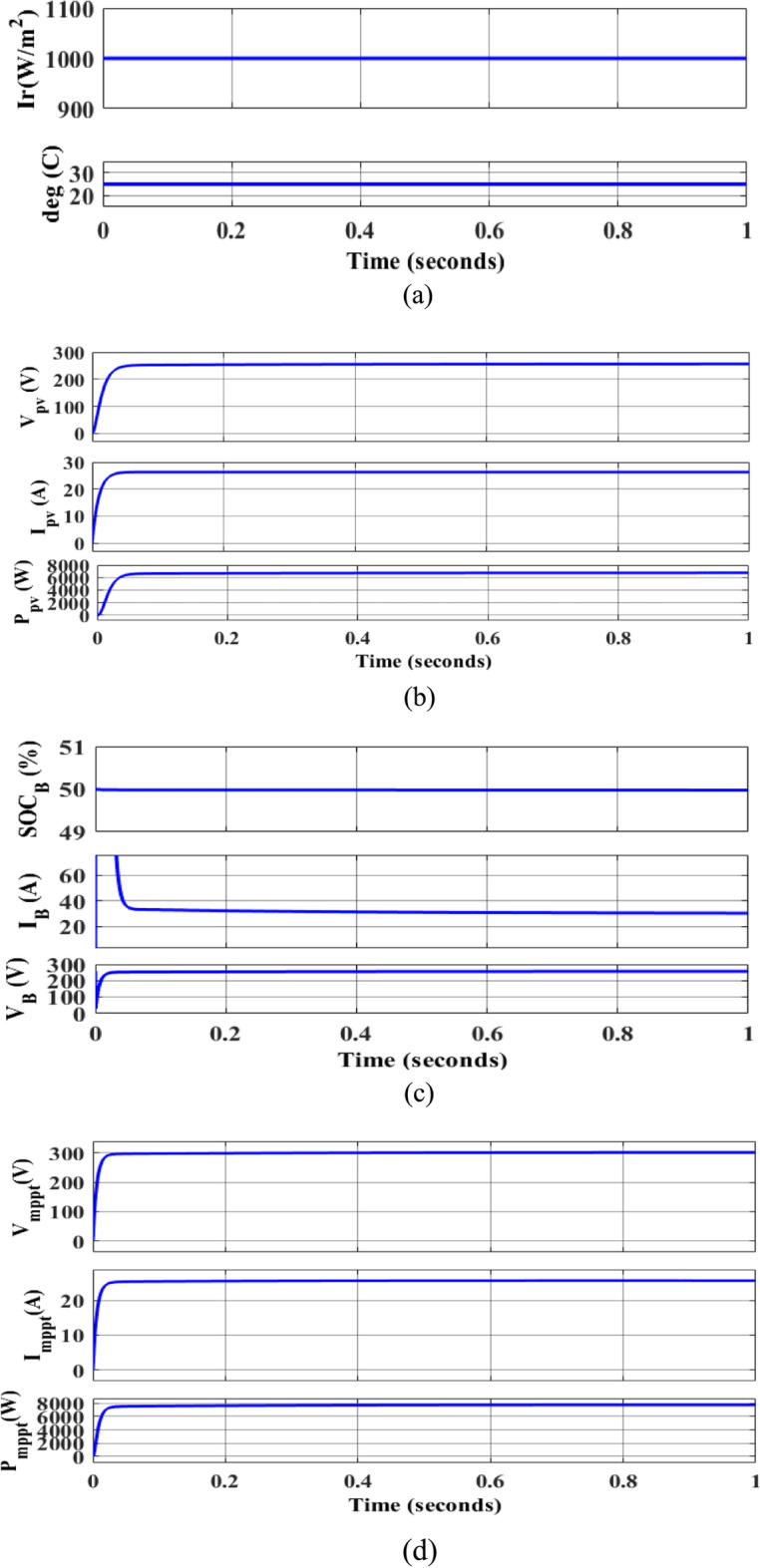

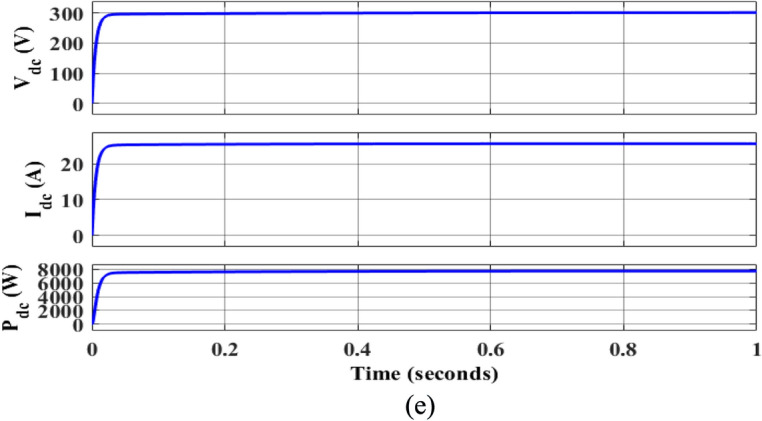
Analysis for different phases in constant stable condition (**a**) irradiance and temperature (**b**) PV analysis for voltage, current, and power performance (**c**) Battery voltage, current, and SOC performance (**d**) MPPT analysis for power, current, and voltage performance (e) DC link current and voltage performance.

In order to expand power extraction under constant irradiance and temperature conditions, this work provides a new MPPT procedure for the grid integrated PV scheme using a novel controller based on improved Dung beetle optimization. As seen in the image, the PV system functioning under typical test settings guarantees the controller environment for performance assessment. Consistently emitting 1000 W/m2, it reaches a temperature of 25 °C. The new MPPT technology, which uses the IDBO algorithm, controls a grid-connected solar system, and Fig. 9

(b) demonstrates the system’s dynamic reaction. A stabilized power output of over 6.76 kW is achieved when the PV voltage swiftly increases to a steady level of about 256 V and the current reaches around 26A. The fact that this behavior is attained in less than 0.01 s shows how quickly the suggested MPPT method can track.

Figure 9c represents the stable response of the battery during the interaction with the PV system. This illustrates three critical parameters over the time interval: SOC, current, and voltage. The SOC remaining stable around 50% indicates that the battery is neither charging nor discharging significantly during this time. The battery shows a sharp drop from the initial position of 65A to a stable operating level around 31.5A. This system quickly stabilizes and demonstrates effective control of charging and discharging. The battery voltage ramps up smoothly to 24 V, which reflects a healthy and controlled voltage regulation. Figure 9 d represents the Simulink response of the PV scheme equipped with an improved MPPT algorithm based on the controller based IDBO algorithm. The system’s stable behavior is depicted as voltage, current, and power that are represented in 1 s intervals. The PV voltage ramps up to 301 V within 0.05 s and remains steady, indicating a rapid and stable voltage regulation under constant irradiance and temperature conditions. The PV current represents a smooth 25.6 A, maintaining the stability to withstand the overshoot. The PV power output reaches close to 7.7 kW, showcasing a successful tracking of the MPP.

The dynamic response of the DC-link in the PV scheme, combined with the sophisticated MPPT controller and inverter, is shown in Fig. 9e. Stability and efficiency are demonstrated by the DC-link voltage, current, and power during a one-second interval. The successful energy transmission from the PV array to the DC bus and efficient voltage management are demonstrated by the DC link voltage’s quick rise and stabilization at 301 V. The DC link current quickly ramps up to nearly 25.6A, which maintains a steady state value. The DC-link power output reaches over 7.735 kW, which represents the system to their MPP. The smooth curve confirms the accuracy of the MPPT and the fine tune controller based optimization.

**Fig. 10:**
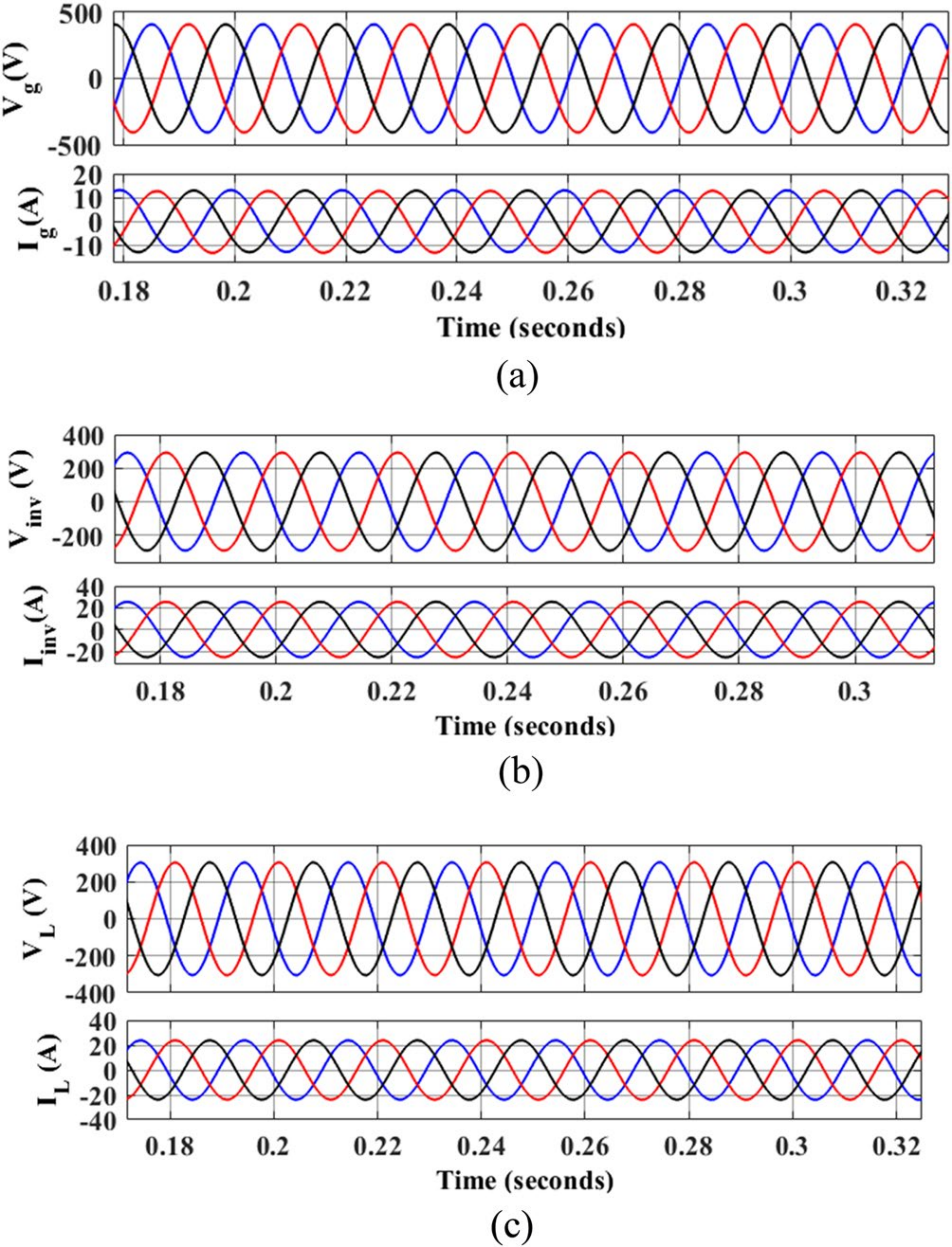
Current and voltage for constant condition (**a**) Grid (**b**) Inverter (**c**) at load.

The grid voltage and grid current waveforms of the grid-connected PV scheme over a one-second period are shown in Fig. 10a.  The presence of an apparent power component at 0.5 Hz is introduced to emulate low frequency artifacts since a practical power grid operates at a nominal frequency of 50 Hz. The waveform analysis corresponds to the inverter output of the grid connected PV scheme integrated with the MPPT and control mechanism represented in Fig. 10b. The system’s dynamic performance is reflected in this sinusoidal waveform. The grid voltage waveform peaks at ± 500 V, indicating that the inverter is efficiently synchronizing with the grid voltage parameters without distortion. This represents a better tuning controller mechanism and stable voltage injection into the grid. The grid current waveform closely follows the voltage waveform in phase and forms the peak current around ± 12A. Low THD and good power quality are indicated by the current’s sinusoidal shape and low harmonic content. The grid-connected PV system, when operated with optimal control, displays the load side voltage and current waveform in Fig. 10c. The inverter demonstrates effective voltage regulates as indicated by the well- defined sinusoidal output waveform with a peak amplitude of approximately ± 230 V. The load current waveform stays in phase and exhibits the smooth sinusoidal pattern peaking at about ± 25A. Near the unity power factor, the suggested phase alignment is functioning, allowing for effective energy delivery to the load.

**Fig. 11:**
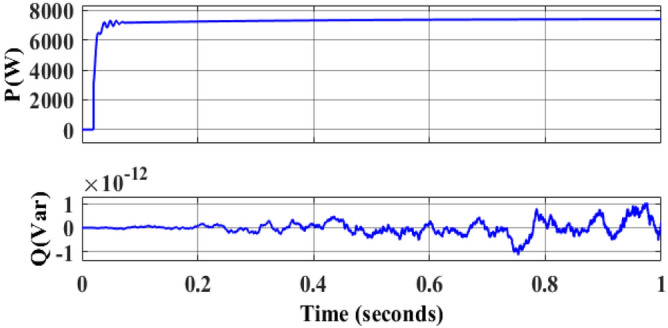
Active and Reactive power performance at constant conditions.

The active and reactive power profiles at the load side of the grid connected PV scheme are represented in Fig. 11. The active power in the top plot rises quickly and reaches a steady state in less than 0.1 s that stabilizing at 7800W. This prompt a seamless reaction depicts how well the control technique maximizes power supply to the load. The reactive power that varies slightly around zero and varies in the range of 10^-12^Var is displayed in the lower plot. The system is operating with the unity power factor, producing just active power with a negligible reactive component.

**Case 2:** Varying Input Irradiance Condition.

**Fig. 12:**
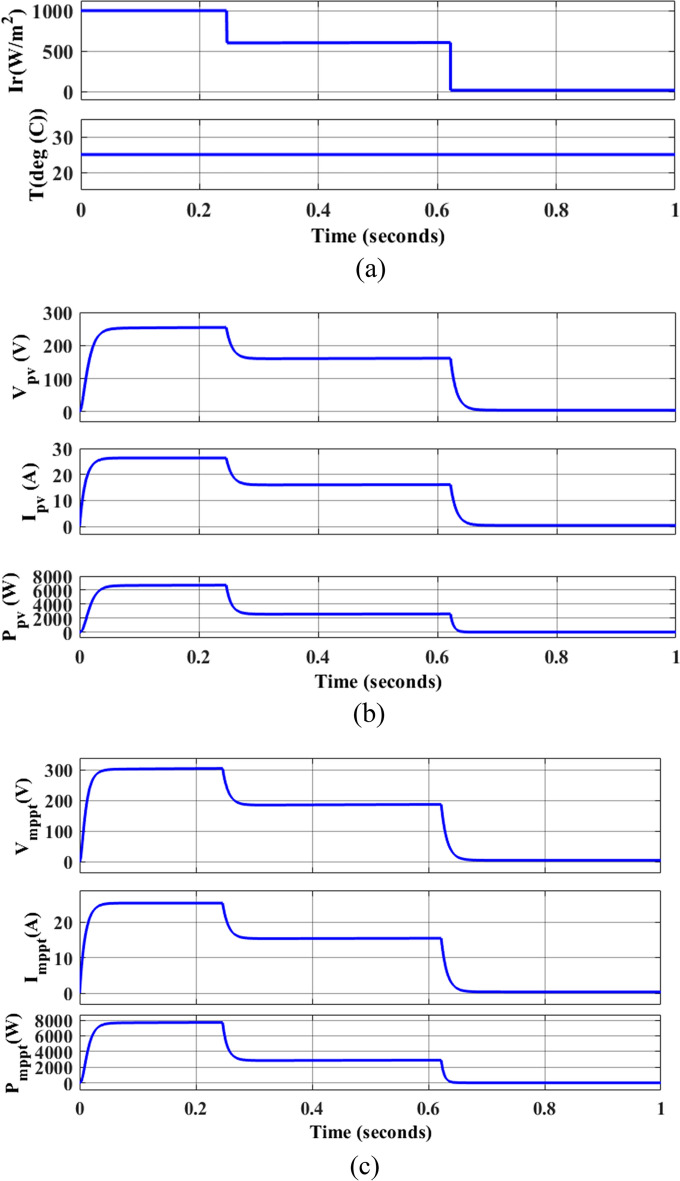

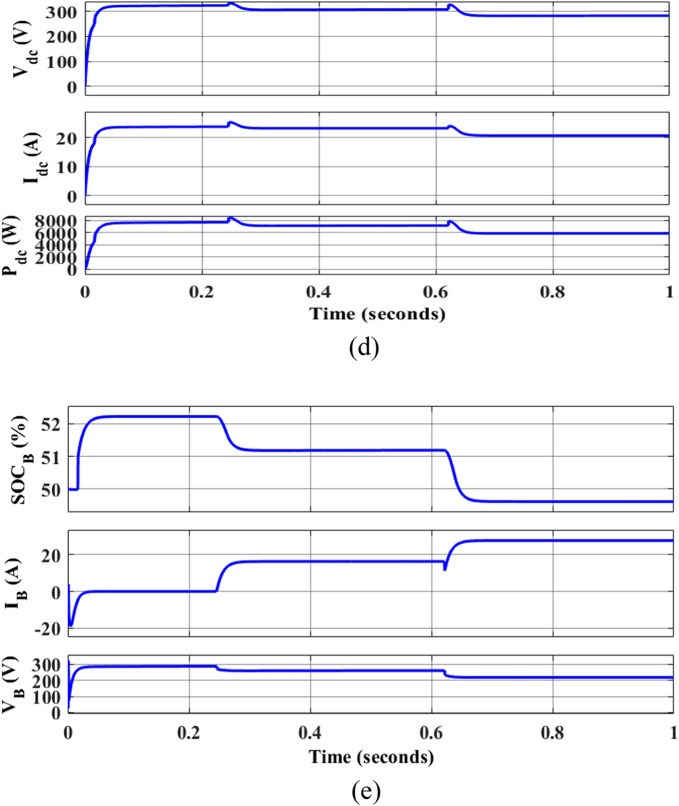
Dynamic analysis for different devices (**a**) irradiance and temperature (**b**) PV analysis for voltage, current, and power performance (**c**) Battery voltage, current, and SOC performance (**d**) MPPT analysis for power, current, and voltage performance (**e**) DC link current and voltage performance.

Figure 12 a represents the dynamic behavior of the irradiance and temperature in different time periods. The irradiance profile simulates rapid variations that are commonly seen in the PV system by gradually decreasing from 1000 W/m2 to 800 W/m2 at 0.3 s and then to 600 W/m2 at 0.6 s. In order to simulate the fluctuating thermal circumstances, temperature profiles are simultaneously varied in discrete increments from 25 °C to 45 °C. In Fig. 12b, the waveform represents the performance of the voltage, current, and power for analyzing the PV system. After

0.2 s, the system reaches 6.46 kW of power, which is progressively reduced to 2.4 kW and ultimately approaches 0 kW at 0.6 kW. The dynamic behavior of the MPPT is represented in Fig. 12c. Similar to PV, the MPPT power also decreases; the voltage, current of 301 V and 25.6A from the MPPT are gradually decreased in the waveform. Figure 12d represents the power, voltage, and current performance of the DC link system. The voltage in the DC link is obtained at 301 V, where there is a small disturbance in the range over a particular time period. The current in the DC link is obtained to be 25.6A and the power is obtained to be 7.735 kW, where there a small disturbances and losses are observed in the waveform. Figure 12d represents the dynamic response of the battery, where the SOC is decreased from 52 to 51% near around 0.2 s. Similarly, the voltage of the battery goes down opposite to that the current that moved up.﻿

**Fig. 13:**
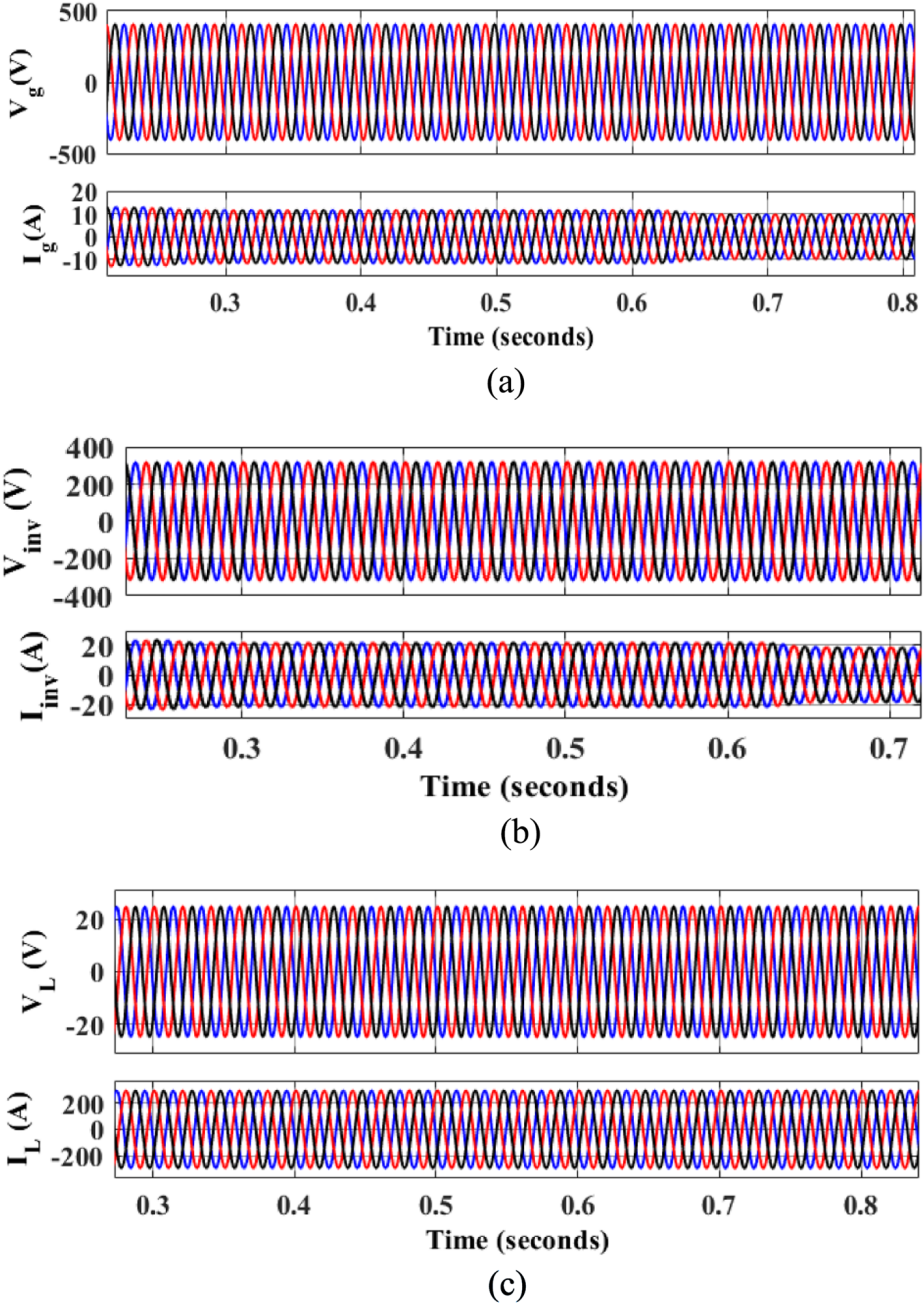
Current and voltage for dynamic condition (**a**) Grid (**b**) Inverter (c) at load.

In Fig. 13, the voltage and current for the different parameters are shown in a dynamic representation. Figure 13a shows the voltage and current waveform of the grid-connected system, with voltage and current varied by ± 408 V and ± 10A, respectively. As seen in Fig. 13b, the inverter’s voltage and current waveform are displayed. In Fig. 13c, the voltage, current waveforms for the load are varied from ± 20 V and ± 20A.

**Fig. 14:**
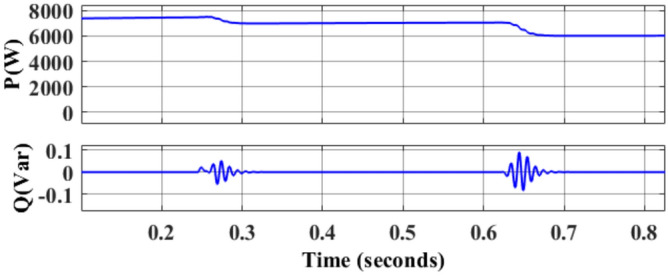
Active and reactive power performance at dynamic irradiance conditions.

Figure 14 exemplifies the active and reactive power performance in the dynamic irradiance condition. A small variation is obtained between the 0.2 s and 0.3 s of the waveform, similarly between the 0.6 s and 0.7 s.

**Fig. 15:**
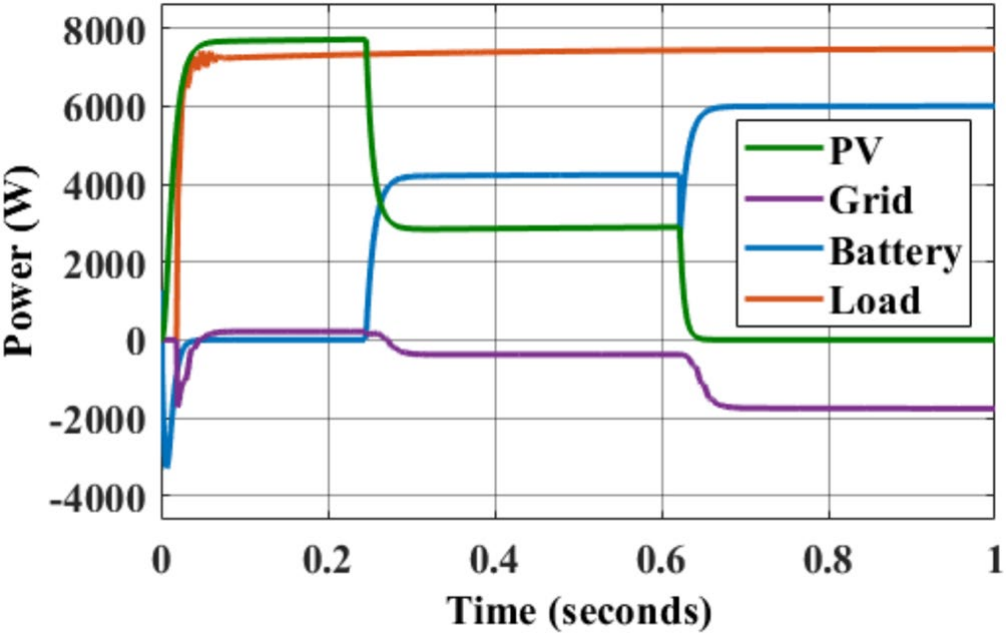
Power analysis at varying conditions.

The dynamic power flow between the PV system, battery, grid, and load under various irradiation circumstances is depicted in Fig. 15. In order to gratify the load demand (orange curve), charge of the battery (blue curve), and the export to the grid (purple curve), the system first produces around 7741.121 W at full irradiance at 1000 W/m2. The PV output falls in proportion to the irradiance when it is lower than 800W/m^2^, which are occurred in 0.3 s. As the battery empties to compensate for the deficiency, the grid resilience begins to increase. The PV production drops precipitously at 0.6 s as the irradiance drops further to 600W/m^2^. Therefore, the grid makes up the remaining difference while the battery accelerates the discharge. The load stays constant during these changes, which indicates that the system can effectively adjust and balance power flow by utilizing the hybrid energy management approach.

**Case 3:** Non-Linear Load Condition.

**Fig. 16:**
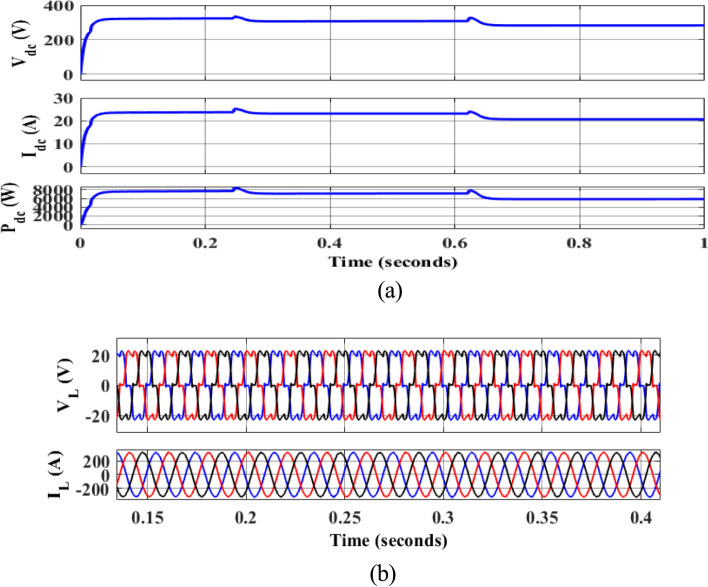
Non-Linear Load Condition for (**a**) DC link, (**b**) load.

Figure 16a represents the DC link performance for voltage, current, and power in the non-linear load condition. There is a variation that is obtained in the system at around 0.2 s and 0.5 s. The non-linearity Fig. 16b load condition is observed to be current at ± 20A and voltage to be at ± 380 V.

**Fig. 17:**
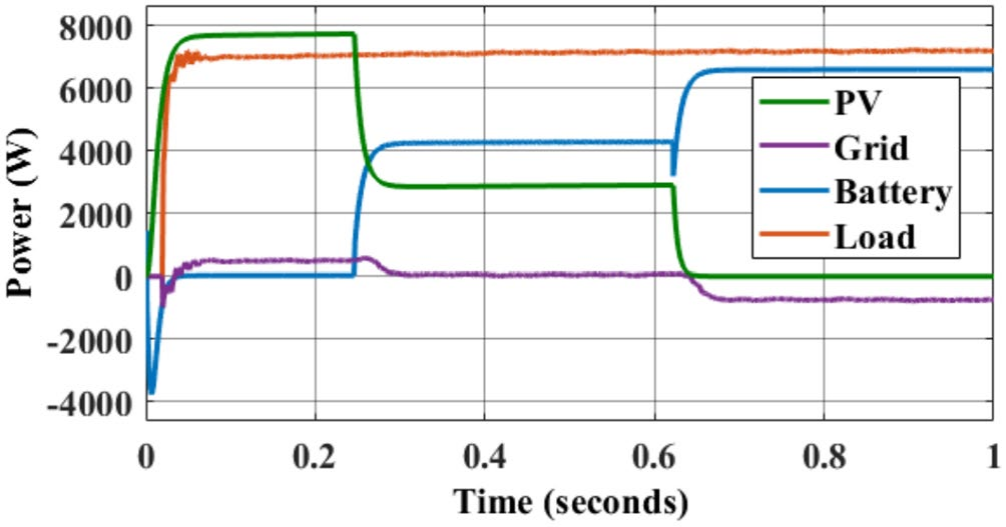
Power analysis at non-linear load.

The power distribution amongst the PV system, battery, grid, and load under a non-linear load situation at the different irradiations that are represented in Fig. 17. The steady orange curve represents the non-linear load that draws a virtually constant power but causes harmonic distortion that needs to be controller with the right filtering and control. The PV system initially provides 1000W/m^2^ irradiance, with the extra power being utilized to charge the battery and export some electricity to the grid. The irradiance decreases at 0.3 s, which lowers the PV production. In order to keep the load supplied, the battery starts to discharge, and the grid starts todeliver the remaining power. Despite the non-linear characteristic of the load, the power control method guarantees smooth adjustment. The PV falls further, and the battery discharges more when there is another irradiance dip at 0.6 s. The load remains unaltered while the grid provides the shortfall. Throughout the whole event, the system controls the distorted load profile caused by the non-linear component without sacrificing stability.

**Case 4:** Varying Load Condition.

**Fig. 18:**
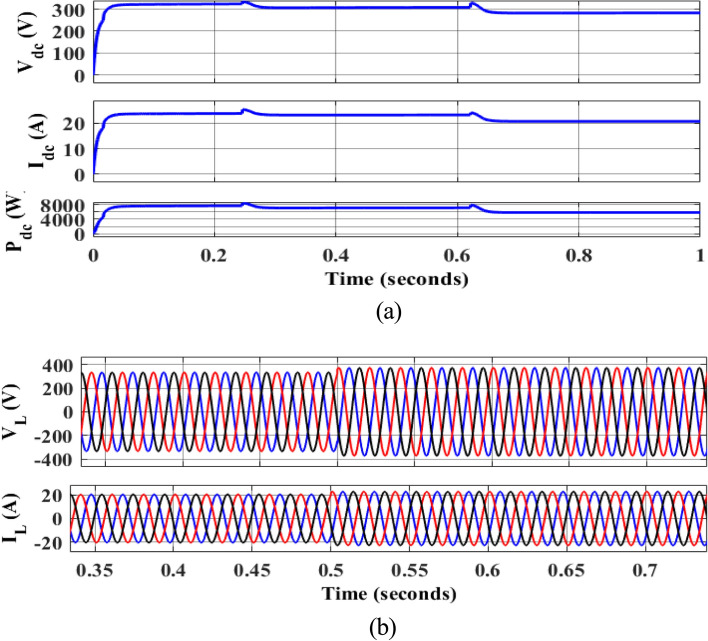
Varying Load Condition for (**a**) DC link and (**b**) load.

Figure 18 represents the varying load conditions for (a) the DC link and (b) the load. In the DC link, the voltage is raised from 330 to 370 V, and the current is raised from 20 to 23A. The power in the DC link system is raised to 6.76 kW.

**Fig. 19:**
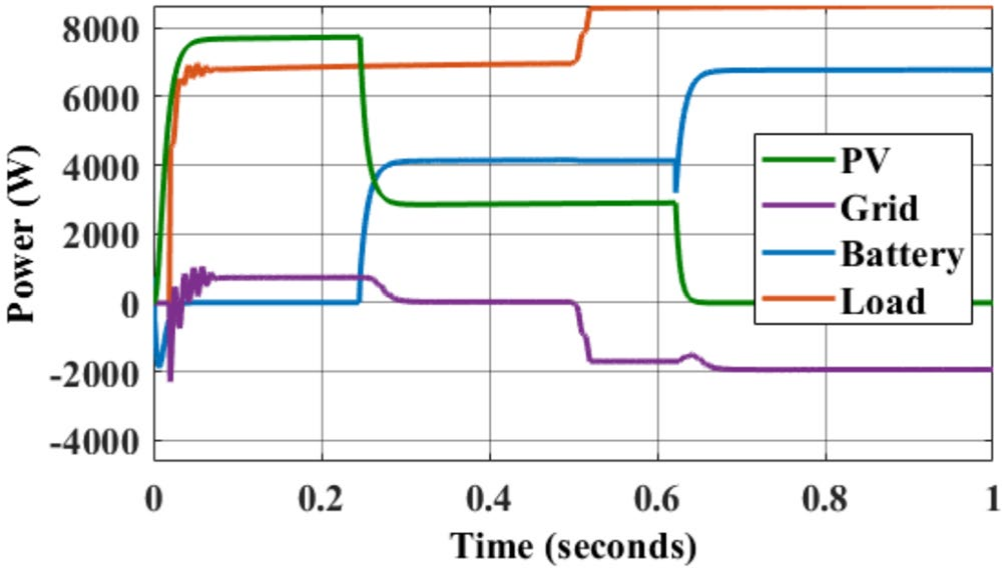
Power analysis for varying load conditions.

Figure 19 represents the power distribution under the non-linear load condition, which maintains a steady 4000 to 6000W. Despite the harmonic reduction, these are effectively managed through filtering and control mechanisms. The PV provides power to charge the battery and export to the grid during the initial 1000W/m^2^ of irradiance. A drop at 0.3 s triggers battery charging and grid support. A further irradiance dip at 0.6 s increases battery discharge, which the grid compensates for this shortfall ensuring an uninterrupted load supply. The system stability under these dynamic conditions highlights the robustness of the integrated power control strategy.

**Case 5:** One Phase Out Fault Condition.

**Fig. 20:**
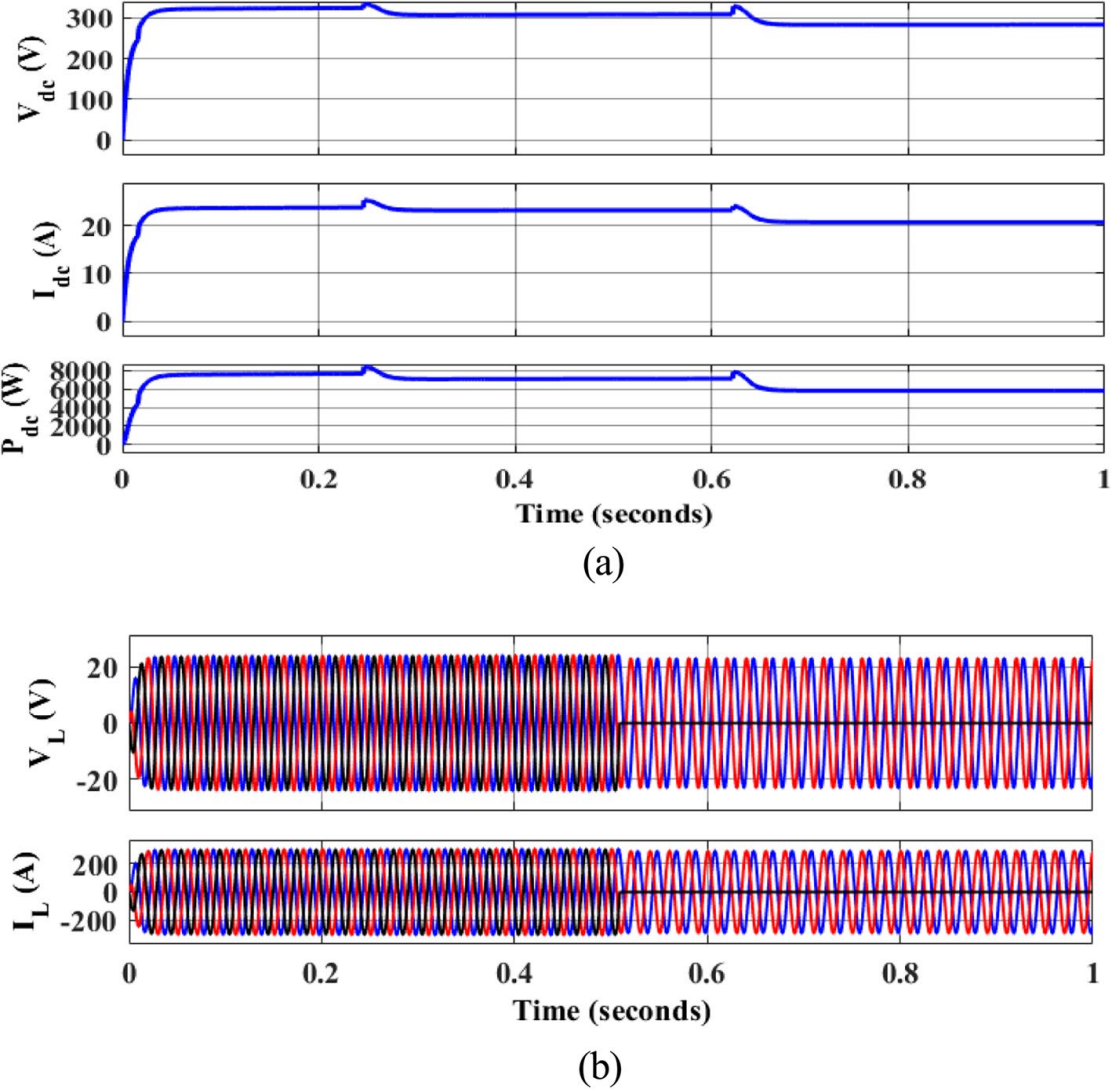
One phase out fault condition for (**a**) DC link, (**b**) load.

Figure 20 represents one phase out fault disorder for (a) the DC link and (b) the load in the proposed method. Here, the voltage is raised to 300 V and the current is raised to 24.9A. The load voltage, current are raised from ± 200 V and ± 20A.

**Fig. 21:**
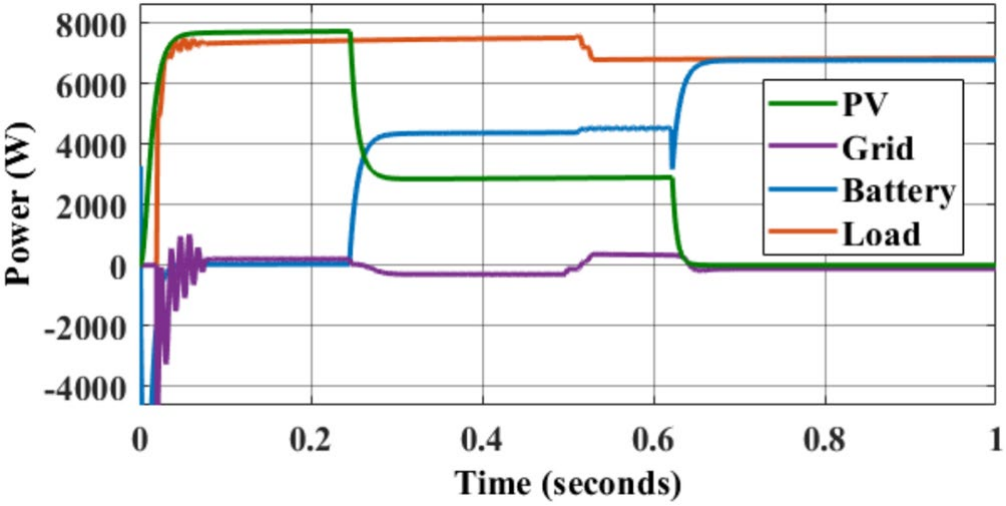
Power analysis for one phase out fault.

Figure 21 depicts the power analysis under a single phase outage fault occurring at 0.1 s. The grid power is purple and exhibits a 4000W, indicating a fault-induced power reversal. As the PV drops from 6000 to 2000W and the battery drains to 2000W, the load stays at 6000W during grid recovery. The fault resilience is demonstrated by the grid stabilizing at 0W post-fault with the PV and battery balancing the load. This demonstrates better system management in energy event of a single phase breakdown.

### Comparative analysis of the proposed IDBO algorithm

To demonstrate the superiority of proposed IDBO based MPPT technique, various parameters are evaluated and compared with Dung beetle optimization (DBO), Piranha Foraging Optimization (PFA), Mother Optimization Algorithm (MOA), Coati Optimization Algorithm (COA), Genetic Algorithm (GA), Grey Wolf Optimization (GWO) and Particle swarm optimization (PSO) algorithms for static and dynamic irradiance and temperature conditions.

#### Comparative analysis of the proposed IDBO algorithm for varying irradiance

Table 3 represents the different parameter values for different algorithms. In this section, irradiance is modified across a distinct time range for each algorithm, and the output section is obtained from the system. Here, different parameters such as steady state value, peak value, peak time, settling time, and overshoot time are being evaluated in this section.

**Table 3:** Different parameter values for different algorithms.

Algori thm	Segment (W/m^2^**)**	Time Range (ms)	Steady- State Value (W)	Peak Value (W)	Peak Time (s)	Settling Time (s)	Overshoot (%)
GA	1000	0–400	7343.983	7352.343	0.397	0.057	0.113
	800	400–700	4758.837	7083.370	0.4	0.432	48.846
	600	700–1000	2675.148	4387.845	0.700	0.735	64.022
MOA	1000	0–400	7182.897	7185.823	0.399	0.057	0.04
	800	400–700	4671.511	7182.048	0.4	0.432	53.741
	600	700–1000	2614.348	4464.17	0.7	0.737	70.756
PSO	1000	0–400	7068.596	7076.856	0.397	0.056	0.1168
	800	400–700	4580.553	6817.613	0.4	0.430	48.838
	600	700–1000	2574.829	4223.467	0.7	0.734	64.029
COA	1000	0–400	7894.789	7903.91	0.397	0.056	0.115
	800	400–700	5115.831	7614.57	0.4	0.431	48.843
	600	700–1000	2875.784	4717.027	0.7	0.735	64.025
GWO	1000	0–400	7435.785	7444.252	0.397	0.057	0.113
	800	400–700	4818.334	7171.897	0.4	0.432	48.845
	600	700–1000	2708.587	4442.714	0.700	0.735	64.023
PFA	1000	0–400	7182.897	7185.823	0.399	0.057	0.040
	800	400–700	4678.311	7186.214	0.401	0.433	53.607
	600	700–1000	2614.35	4682.356	0.701	0.741	79.102
DBO	1000	0–400	7524.661	7527.388	0.392	0.053	0.036
	800	400–700	4901.686	7519.148	0.4	0.426	53.399
	600	700–1000	2738.678	4905.879	0.701	0.736	79.133
Propos ed	1000	0–400	7723.613	7741.121	0.397	0.049	0.226
	800	400–700	5071.956	7185.893	0.4	0.424	41.678
	600	700–1000	2854.795	5076.061	0.701	0.736	77.808

**Fig. 22:**
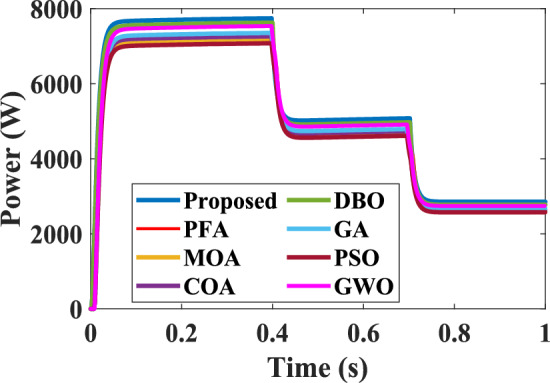
MPPT for varying irradiance conditions.

The output power response of the suggested MPPT method under varying irradiance is shown in Fig. 22. In comparison to other algorithms, the suggested hybrid approach shows better tracking capacity and faster convergence during the irradiance drop at 0.4 s and 0.7 s. The proposed method consistently maintains the highest power generation, whereas the MOA and PFA show slower responses and lesser power extraction. COA and DBO perform moderately better but are clearly outperformed by the proposed strategy in both speed and stability.

#### Comparative analysis of the proposed IDBO algorithm for varying temperature

Table 4 represents comparison of different algorithms for various parameters in varying temperature condition. Here the temperature is reduced to 35 C̊ and 45 C̊ at 0.4 s and 0.7 s, and output is analyzed for different algorithm such as PFO, MOA, COA, DBO, PSO, GA and GWO algorithm.

**Table 4:** Different algorithms in varying temperatures for different parameters.

Algorithm	Temperature	Steady State (W)	Peak Time (s)	Settling Time (s)	Overshoot percentage
GA	25 °C	6176.165	0.093	0.197	7.971
	35 °C	5254.106	0.4	0.437	13.205
	45 °C	4430.062	0.701	0.789	18.627
MOA	25 °C	5770.595	0.088	0.215	8.219
	35 °C	4934.084	0.4	0.406	16.734
	45 °C	4144.382	0.703	0.795	19.078
PSO	25 °C	6064.367	0.094	0.197	7.921
	35 °C	5158.544	0.4	0.438	13.212
	45 °C	4349.417	0.702	0.790	18.629
COA	25 °C	6400.871	0.093	0.213	7.961
	35 °C	5445.156	0.4	0.437	13.207
	45 °C	4591.133	0.701	0.790	18.627
GWO	25 °C	6625.581	0.093	0.197	7.950
	35 °C	5636.207	0.4	0.438	13.208
	45 °C	4752.201	0.702	0.790	18.628
PFA	25 °C	5895.644	0.093	0.213	7.96
	35 °C	5032.481	0.4	0.41	12.964
	45 °C	4243.596	0.701	0.789	18.611
DBO	25 °C	6737.757	0.093	0.213	7.9613
	35 °C	5731.744	0.4	0.437	13.207
	45 °C	4832.771	0.701	0.79	18.627
Proposed	25 °C	7007.978	0.088	0.216	8.316
	35 °C	5991.688	0.4	0.406	16.74
	45 °C	5032.671	0.703	0.795	19.079

**Fig. 23:**
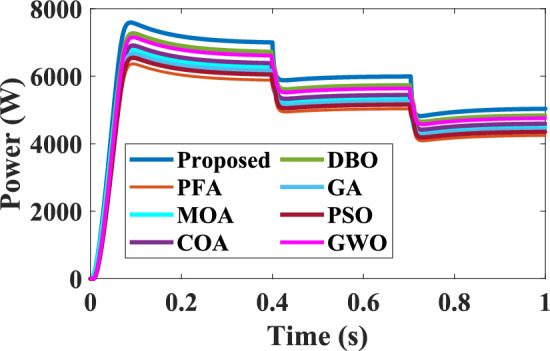
MPPT comparison for varying temperature.

The output power response of the suggested MPPT method under dynamic temperature conditions is shown in Fig. 23. Under the fluctuating temperature condition, the proposed method demonstrates improved adaptability. Due to the inherent temperature sensitivity of the PV module, all the methods experience a temporary drop in output power. However, the proposed MPPT approach stabilizes more rapidly at the true MPP. While the COA and DBO provide performance, they still lack the efficiency and robustness of the suggested approach, while MOA exhibits the slowest recovery and the worst steady state performance among the options.

#### Analysis of error parameters

Table 5 represents the error parameters of different algorithms. In this section the values for different algorithm for different parameter error are taken such as Mean square error (MSE), Root mean square error (RMSE) and Mean absolute error (MAE) are taken.

**Table 5:** Error parameters of different algorithms.

Algorithm\error	MSE	RMSE	MAE
GA	0.088	0.296	0.222
MOA	0.086	0.293	0.226
PSO	0.082	0.287	0.221
COA	0.081	0.285	0.216
GWO	0.081	0.285	0.215
PFA	0.077	0.278	0.218
DBO	0.059	0.244	0.203
Proposed	0.046	0.215	0.175

### Comparative analysis of THD

**Fig. 24:**
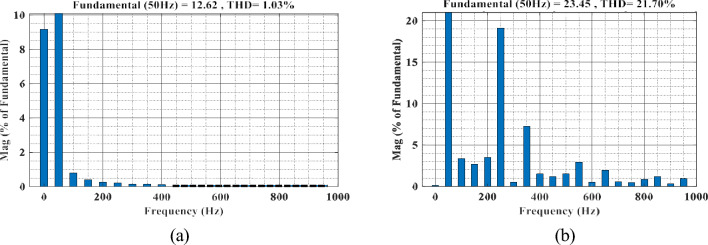
Analysis of THD for (**a**) Grid (**b**) Non-linear Load.

Figure 24 represents THD analysis reveals the minimal harmonics on the grid side, confirming effective harmonic suppression by the proposed control strategy. As a result of its non- linear character, the load side displays a pronounced harmonic distortion. In spite of this, the grid’s electricity quality remains high. This proves that the controller can separate grid disturbances from load disturbances. This performance validates the efficacy of the proposed control strategy in achieving substantial harmonic suppression while maintaining high power quality at the point of common coupling, which exhibits outstanding compliance with IEEE 519 criteria for grid-side current harmonics.

**Fig. 25:**
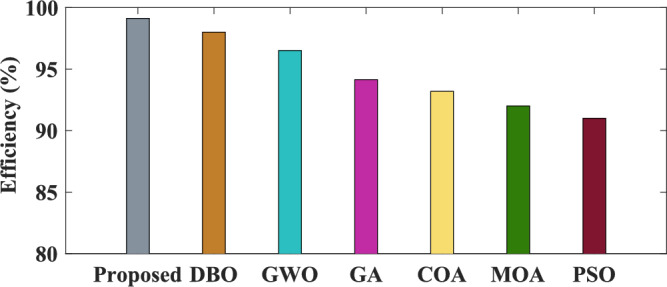
Comparison of efficiency for different algorithms.

Figure 25 represents the efficiency comparison of different algorithms; the suggested scheme attains an efficacy of 99.12% which is greater than other methods. The algorithm method achieves DSO efficiency of 98%, GWO of 96.51%, GA of 94.14%, COA of 93.2%, MOA of 92.01%, and PSO of 91%. The substantial improvement in efficiency confirms the effectiveness of the suggested methodology in optimizing performance, which highlights the robustness and capability in achieving the near optimal result with minimum losses.

#### Comparative analysis of THD

**Fig. 26:**
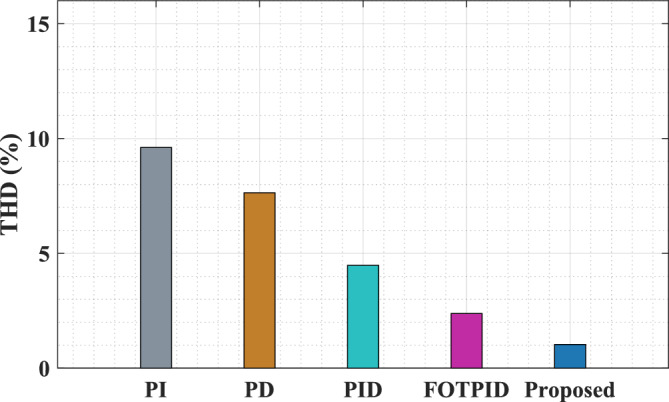
THD of different controllers.

Figure 26 represents THD values for the control techniques. The proposed method achieves 1.03%, PD obtains 7.64%, PID obtains 4.48%, FOTPID obtains 2.38%, and PI gives 9.61%. Because of its fundamental proportional integral action, the PI controller has the greatest THD (9.61%), which indicates the weak capacity to suppress the harmonics. The PD controller decreases the THD to 7.64%, and the PID controller increases it to 4.48%.

#### Convergence curve analysis

**Fig. 27:**
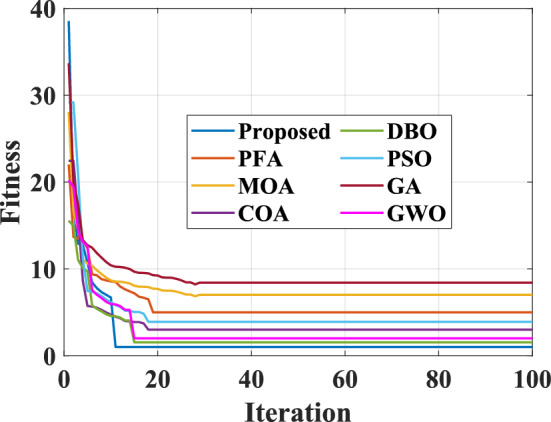
Convergence waveform for different algorithms.

The suggested approach suggests a rapid convergence by giving a fitness value of 1 after 11 iterations, as shown in Fig. 27, which displays the convergence waveform for various algorithms. While the COA and DBO converge more slowly to attain the value around 3 to 1.5 by 15 and 18 iterations, PFA and MOA exhibit an early drop around 5 to 7, which stabilizes after 19 and 29 iterations. Here, the PSO attains a drop of 18 iterations in 3.9 fitness value, GA attains a drop of 15 iterations in 2 fitness value, which is closer to the proposed, and GWO attains a drop of 29 iterations in 8.4 fitness value. The proposed approach performs better than the other, with the quickest and biggest fitness decrease, which indicates higher optimization potential and improves the search tactics.

#### Discussion

The performance evaluation of the proposed IDBO-based hybrid control strategy demonstrates its effectiveness under multiple operating scenarios. Under standard test conditions, the PV system exhibits rapid stabilization in voltage, current and power. The smooth DC-link regulation and stable battery SOC response further validate the coordinated energy management. The inverter, grid, and load waveforms maintain sinusoidal characteristics indicating effective synchronization and high-quality power injection. The proposed method maintains superior tracking compared to GA, PSO, MOA, COA, GWO, PFA and conventional DBO. The comparative results show improved steady-state power, reduced settling time, and better overshoot control across different irradiance segments and temperature levels. The error analysis confirms enhanced accuracy, as reflected by lower MSE, RMSE, and MAE values. These outcomes highlight the robustness of the improved exploration–exploitation balance in the IDBO algorithm. Despite the presence of harmonic, the grid-side current remains minimally distorted, confirming effective harmonic isolation and compliance with IEEE 519 standards.

The comparative THD analysis shows that the proposed controller significantly outperforms conventional PI, PD, PID, and FOTPID controllers. During the one-phase-out fault condition, the system successfully maintains load continuity through coordinated power sharing.

## Conclusion

The work presented an improved hybrid inverter control framework gor a grid connected PV with the IDBO algorithm. The IDBO algorithm with the Fuch chaotic mapping effectively mitigates the limitation of the conventional dung beetle optimizer. The integration of the FOTPIDn(1 + PD) controller with the filtering mechanism strengthens voltage regulation, harmonic suppression and dynamic system stability. The simulation result confirms the proposed control method improves the power quality, ensures rapid dynamic response in irradiance and temperature variation and maintains stable DC link voltage. The output gives 1.08% THD with a steady-state value of 7723.613 W, a peak value of 7741.121 W, a peak time of 0.397 s, a settling time of 0.049 s, and an overshoot of 0.22%. The proposed optimization obtained optimal values within 11 iterations, which shows better convergence than existing algorithms. The study focuses primarily on grid-connected PV systems under specific operating scenarios. Extreme grid disturbances, large-scale PV farm integration, and long-term operational reliability were not considered. Future research might investigate leveraging an embedded system platform to implement the suggested IDBO algorithm based controller for MPPT in real time hardware. The strategy may expand to include a hybrid renewable energy system that combines a fuel cell, wind power large scale PV farm that is connected to the smart networks.

## Data Availability

All data generated or analyzed during this study are included in this published article.
